# Internet Searches for Medical Symptoms Before Seeking Information on 12-Step Addiction Treatment Programs: A Web-Search Log Analysis

**DOI:** 10.2196/10946

**Published:** 2019-05-03

**Authors:** George Nitzburg, Ingmar Weber, Elad Yom-Tov

**Affiliations:** 1 Teachers College Columbia University New York, NY United States; 2 Social Computing Department Qatar Computing Research Institute Hamad Bin Khalifa University Doha Qatar; 3 Microsoft Research Redmond, WA United States; 4 Microsoft Research Herzeliya Israel; 5 Faculty of Industrial Engineering and Management Technion - Israel Institute of Technology Haifa Israel

**Keywords:** alcohol use disorder, substance use disorder, 12-step programs, brief intervention, brief physician advice, anonymized internet search log data

## Abstract

**Background:**

Brief intervention is a critical method for identifying patients with problematic substance use in primary care settings and for motivating them to consider treatment options. However, despite considerable evidence of delay discounting in patients with substance use disorders, most brief advice by physicians focuses on the long-term negative medical consequences, which may not be the best way to motivate patients to seek treatment information.

**Objective:**

Identification of the specific symptoms that most motivate individuals to seek treatment information may offer insights for further improving brief interventions. To this end, we used anonymized internet search engine data to investigate which medical conditions and symptoms preceded searches for 12-step meeting locators and general 12-step information.

**Methods:**

We extracted all queries made by people in the United States on the Bing search engine from November 2016 to July 2017. These queries were filtered for those who mentioned seeking Alcoholics Anonymous (AA) or Narcotics Anonymous (NA); in addition, queries that contained a medical symptom or condition or a synonym thereof were analyzed. We identified medical symptoms and conditions that predicted searches for seeking treatment at different time lags. Specifically, symptom queries were first determined to be significantly predictive of subsequent 12-step queries if the probability of querying a medical symptom by those who later sought information about the 12-step program exceeded the probability of that same query being made by a comparison group of all other Bing users in the United States. Second, we examined symptom queries preceding queries on the 12-step program at time lags of 0-7 days, 7-14 days, and 14-30 days, where the probability of asking about a medical symptom was greater in the 30-day time window preceding 12-step program information-seeking as compared to all previous times that the symptom was queried.

**Results:**

In our sample of 11,784 persons, we found 10 medical symptoms that predicted AA information seeking and 9 symptoms that predicted NA information seeking. Of these symptoms, a substantial number could be categorized as nonsevere in nature. Moreover, when medical symptom persistence was examined across a 1-month time period, a substantial number of nonsevere, yet persistent, symptoms were identified.

**Conclusions:**

Our results suggest that many common or nonsevere medical symptoms and conditions motivate subsequent interest in AA and NA programs. In addition to highlighting severe long-term consequences, brief interventions could be restructured to highlight how increasing substance misuse can worsen discomfort from common medical symptoms in the short term, as well as how these worsening symptoms could exacerbate social embarrassment or decrease physical attractiveness.

## Introduction

### Background

Alcohol use disorder (AUD) exerts a heavy burden on psychiatric and medical facilities, which has led to early identification and intervention efforts with problem drinkers who are engaging in regular risky drinking patterns but do not yet have physical dependence on alcohol or an immediate medical need for abstinence. For many problem drinkers, the first point of contact often occurs at routine office visits to primary care physicians, where roughly 10%-20% of primary care patients are found to be drinking alcohol at hazardous levels [1-3]. Risky drinking patterns (such as binge or regular heavy drinking) have been linked with numerous adverse medical consequences [4-9] including numerous cancers [5], heart disease and stroke [10-13], pneumonia [14], tuberculosis [15], epilepsy [16], diabetes [17], pancreatitis [18-21], and liver disease [22]. Although the abovementioned conditions are among the more severe long-term medical consequences of alcohol, heavy alcohol consumption has been implicated in the onset or exacerbation of over 100 diseases and conditions [23]. Since primary care office visits can often be prompted by the need to address medical symptoms partially or can fully arise from heavy drinking patterns, such visits offer an opportunity to motivate problem drinkers toward alcohol-reduction treatment options.

To help reduce drinking among their patients, from hazardous levels down to low-risk levels, physicians have made efforts to integrate minimal treatment protocols into their routine care (via face-to-face or electronic delivery), known as brief intervention (BI) [24,25]. The largest venue for BI, which follows after The Substance Abuse and Mental Health Services Administration’s largest-of-its-kind federally funded Screening, Brief Intervention, and Referral to Treatment (SBIRT) initiative, is primary care clinics and emergency rooms. SBIRT has been a vital source of early identification of alcohol and drug use disorders, which has so far screened and offered BI or treatment referral in 29 US states [26]. SBIRT programs have demonstrated effectiveness with drug and alcohol reductions at 6 months postintervention [27], along with clinically and statistically significant improvements in physical and mental health as well as social, legal, housing, and employment outcomes [28]. In addition to expanding the scale of the existing SBIRT program, identifying ways to further maximize BI’s effectiveness is also of critical importance to SBIRT.

One largely unexplored avenue for enhancing BI’s personalized feedback for more severe cases is to evaluate which negative medical consequences most contribute to information seeking for abstinence-based treatment. Twelve-step programs remain the most utilized form of substance use disorder (SUD) treatment [29,30]. Specifically, BI’s health risk information could be improved by understanding which of the >100 alcohol-implicated diseases and conditions are most likely to motivate severe cases to consider 12-step treatment options. Many potential improvements may be possible regarding this health risk information.

In particular, many of the medical consequences emphasized in BI occur over the long term (eg, cancer). However, meta-analytic evidence has shown that individuals who engage in addictive behaviors make more impulsive decisions, have greater difficulty delaying gratification for greater rewards, and tend to discount delayed rewards or other reinforcers more quickly than healthy controls [31,32]. Thus, the actual and perceived speed of reward or reinforcer delivery is particularly critical to motivation in patients with AUD, and as a result, they may be more motivated by less chronic but more immediately discomforting and socially embarrassing symptoms such as gastrointestinal distress. Thus, BI’s health risk information could potentially be improved by focusing on an increased likelihood of experiencing specific sets of immediately discomforting and socially embarrassing symptoms rather than a risk of diagnosis with various medical diseases, some of which occur in the long term (eg, cancer). Reorganizing BI’s health-risk information around the medical symptoms that can be empirically shown to motivate treatment seeking holds the potential to increase BI’s efficacy for the more severe cases often seen in primary care settings.

### Prior Work

Analysis of large-scale anonymized Web-search log data offers a promising new method for identifying medical symptoms that are most predictive of subsequent seeking of 12-step treatments. Recent analyses of subtle and temporal changes in medical symptom Web-search queries have been able to predict later medical events, including influenza [33], pancreatic and breast cancers [33,34], and adverse side effects of medications [35]. Such analyses can be readily adapted from the prediction of dichotomous health events (eg, search log evidence of having cancer versus not having cancer) to the prediction of dichotomous behavioral health care choices (eg, search log evidence of interest in 12-step treatment versus no such treatment). Although many researchers investigating online patterns in psychiatric disorders and SUD have opted to examine social media activity rather than search engine queries [36-48], Web-search log data analysis is particularly well suited to the study of alcohol-related medical symptoms. Past work has shown that medical health information seeking most often takes place using search engines, especially when medical symptoms may be perceived as potentially connected to stigmatized conditions (eg, symptoms perceived to be the possible negative medical consequences of addiction) [49]. It is most likely due to the associated stigma that regional variation in the interest in the topics of alcohol and Alcoholics Anonymous (AA) on Facebook show inconsistent patterns with respect to regional variation in the rates of alcohol abuse [50]. Due to its anonymous nature, aggregate and anonymous Web-search volume from Google Trends has also been used to study the link between macroeconomic conditions and problem drinking and has shown that a 5% rise in unemployment is followed by an approximate 15% increase in alcoholism-related searches in the next 12 months [51]. In addition, using data from Google Trends, other researchers have assessed the relationship between different legal statuses of marijuana and the level of search interests for “dabbing,” the vaping of high-potency marijuana concentrates [52].

At the individual level, anonymized Web-search log data offer an ecologically valid and representative sampling of individuals who are likely to show up at primary care clinics and disclose their alcohol-related medical symptoms to a physician when prompted. A recent systematic review found that individuals experiencing acute or conspicuous medical symptoms searched for online health information prior to seeking real-world medical care. These individuals were also subsequently more persuaded to see a physician than those who did not do a Web search, endorsing less embarrassment and concern about bothering their physician with a trivial complaint [53]. Search log data analysis is also well suited to reorganizing BI’s brief advice by a physician, regarding the medical consequences of alcohol misuse, as the same type of analysis has already helped inform how online cancer information may be reorganized to best address the distinct needs of patients and their caregivers when patients are at different levels of disease progression [54]. Taken together, past work suggests that Web-search log data analysis is the best-suited technique to provide unique insights into which negative medical consequences of alcohol misuse are most persuasive for motivating interest in the 12-step treatment.

### Goal of the Study

The present study used anonymous internet search log data to better understand how seeking medical symptom information contributed to seeking AA and narcotics anonymous (NA) treatment information, including searches for treatment locators. We examined anonymous Microsoft Bing search data to identify individuals that graduated from using Bing search to investigate their medical complaints (which may be potentially alcohol related) to using Bing search to seeking AA or NA information or finding AA or NA meetings. We then examined which of these medical symptom searches most increased the likelihood of conducting either of these two types of Bing searches. Such data can help inform BI-personalized feedback to enhance acceptance of SBIRT’s treatment referrals for patients spanning the broader range of alcohol and drug use severity levels often seen in primary care physicians’ offices.

Of note, we chose to include NA-related queries in our primarily AUD-focused study since alcohol and drug use disorders are highly comorbid, AA meetings are far more ubiquitous than NA meetings, people with drug use disorders are not typically turned away from AA meetings after revealing their drug of choice is not alcohol, these two types of 12-step programs share many characteristics, and both meeting types can often have mixed attendance [55]. Given such comorbidity and cross-attendance, our online search data analysis was unable to reasonably estimate users’ diagnostic groups, and we instead opted to separately analyze all the various combinations and permutations of AA- and NA-related queries to gauge the convergence/divergence of symptom profiles that preceded them.

### Hypotheses

We proposed the following hypotheses:

1. The medical symptom queries motivating subsequent seeking of information on 12-step programs would be nonsevere but immediately discomforting and socially embarrassing.
2. Symptoms or conditions considered severe or medically dangerous would not require persistence over time to induce queries for the 12-step programs, with individuals’ taking near-immediate action to seek out information on the 12-step programs soon after experiencing these symptoms.
3. Symptoms or conditions considered common or nonsevere would require persistence over time to induce queries about the 12-step programs, with individuals only taking action to seek out information on the 12-step programs after a period of enduring discomfort from these symptoms.

## Methods

### Data

We extracted all queries made by people in the United Stated on the Bing search engine from November 2016 to July 2017 (inclusive). Each query contained an anonymous user-identifier tracked via a browser cookie, the time and date of the query, the text of the query, and the Web address of pages clicked by users in response to the answers shown to them. Although Bing’s US market share is currently estimated at ~24% [56], past work has found it to be representative of the US population [57,58] and has shown that Bing is a viable data source even in the absence of other search engines. This study was approved by the Institutional Review Board of the Technion, Israel Institute of Technology.

### Analysis

#### Identifying Queries of People Seeking Information on Alcoholics Anonymous and Narcotics Anonymous

We then found all queries made by 50 or more people, which resulted in a click to one or more of the 193 pages. There were a total of 100 such queries (eg, “alcoholic anonymous meeting directory”).

#### Identifying the Target Population

Based on the queries identified, we defined two target groups (ie, two 12-step information-seeking samples). The first larger overall sample comprised all 11,784 people who made an AA- or NA-seeking query during July 2017 and who did not make such queries in any of the preceding months (November 2016 to June 2017). The second sample, a subset of the first, included only those people who specifically queried for AA or NA meeting locators by seeking queries that also contained the term “meeting.” A total of 3820 people were included in this subsample.

Our overall sample consisted of a total of 10,522 people who sought AA information, 1022 people who sought NA information, and 240 people who sought both AA and NA information. Our subsample (whose first 12-step–related search was for AA or NA meeting information) consisted of 2324 people who sought information specifically on AA meetings, 501 people who sought information specifically on NA meetings, and 115 people who sought information on both types of meetings.

Of note, we did not require a medical symptom query to have been conducted in order for the query to be included in our comparison group. In other words, our overall sample also included people who searched for information on the alcohol or drug 12-step program without ever having queried a medical complaint.

#### Identifying Symptoms Correlated With Seeking Treatment Information

Queries were deemed to contain medical symptoms if their text included a symptom or a synonym thereof, as provided in the list of 195 symptoms and their synonyms in the study by Yom-Tov and Gabrilovich [35]. Colloquial symptoms were mapped to their medical symptom wording, which is why medical terminology descriptors appear in our data (eg, a search query for “sweating” would be mapped onto the more medical descriptor “diaphoresis”). This list included medical symptoms that were indicative of both medical and psychological distress (eg, fever and cough as well as anxiety and depression). The list also included both symptoms that would be immediately discomforting as well as distant or long-term diagnoses (eg, gastrointestinal distress versus cancer).

**Figure 1:**
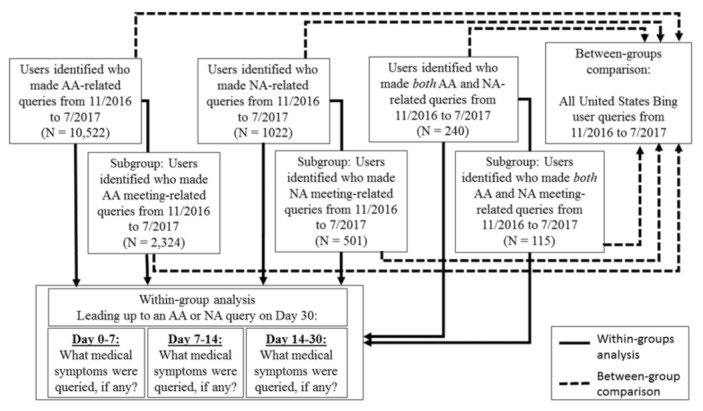
Procedure for data collection, filtering, and analysis with a between-group comparison and time-lagged within-group comparison of medical symptom queries. NA: narcotics anonymous; AA: alcoholics anonymous.

We compared the symptoms prior to seeking information on the 12-step programs in two ways: First, we compared the entire population of Bing users in the United States. Second, we performed a within-group comparison, taking into account the temporal sequence of searches. When examining these temporal sequences, we categorized “symptoms with persistence in time” as symptoms that were repeatedly queried across any two of our three measurement points (measurement points at 0-7, 7-14, and 14-30 days prior to a 12-step–related search); symptoms were defined as persistent when they were queried during both the 0-7 day and 14-30 day time lags, during both the 7-14 day and 14-30 day time lags, or during both the 0-7 day and 14-30 day time lags. A full schematic of procedures for data collection and filtering as well as the between-group comparison and the time-lagged within-group analysis of medical symptom queries for identified users are provided in Figure 1.

For the first analysis, we calculated the probability that users in the treatment population will ask about each symptom in their queries, divided by the same probability for all Bing users in the United States. For the second analysis, we defined a window of time prior to the first query seeking information on the 12-step programs for each person. We then constructed a 2 × 2 table for each symptom (Table 1) and calculated the Chi-square score of this table. We only retained symptoms where the probability of asking about a symptom was greater in the time window than all previous times and where the Chi-square score was statistically significant at *P*<.05 with Bonferroni correction (*P*=.05/[152*3]=.00011). This approach identified cases where there was a statistically significant increase in the queries for a symptom in the days prior to the first query seeking information about treatment.

**Table 1:** A 2 × 2 table for each symptom to evaluate the change in queries for the symptom in each population.

Queries made in T days before seeking information on the 12-step programs	Number of people who queried about the symptom	Number of people who did not query about the symptom
Yes	A	C
No	B	D

## Results

When comparing the data of all the people who queried a medical symptom in the United States, our analysis of 195 medical symptoms revealed that 10 medical symptoms predicted AA information seeking and 9 symptoms predicted NA information seeking. Results also identified 10 medical symptoms that predicted subsequent querying for an AA meeting and one symptom (bloating) that predicted subsequent querying for an NA meeting. Of these symptoms, a substantial number could be categorized as nonsevere in nature (Textbox 1). Moreover, when medical symptom persistence was examined across a 1-month time period, a substantial number of nonsevere, yet persistent, symptoms were identified (Table 2).

We first hypothesized that given the evidence of heightened levels of delayed discounting in SUD populations compared to controls [31,32], more immediately discomforting and socially embarrassing medical symptoms and conditions would be most prevalent for those who subsequently search for information on the 12-step treatment program. This first hypothesis was only partially confirmed. As hypothesized, compared to all other Bing search users in the United States, the probability of a user querying about AA or NA, in general, or the meeting information was significantly increased by earlier queries for nonsevere symptoms and conditions such as bloating, sweating, hives, heartburn, dizziness, bulging eyes, impotence, and back ache. However, several severe symptoms significantly increased the likelihood of subsequent AA or NA queries, although it was unclear if they were primarily medical, psychiatric, or related to substance withdrawal.

We also hypothesized that the symptom queries that were significantly linked to immediate/near-immediate seeking of 12-step information would be severe or medically dangerous; severe symptoms would not require persistence over time to produce treatment motivation. Surprisingly, this second hypothesis was not confirmed. No medical symptom queries were significantly linked to immediate seeking of NA information or concurrent NA and AA information (eg, co-occurring drug and alcohol addiction treatment). In addition, the symptom queries that were significantly linked to the immediate seeking of AA information primarily consisted of common and nonsevere medical conditions such as cramp, rash, toothache, and dry mouth (the exceptions were hallucination and phobia, which could also be psychiatric or related to withdrawal).

Last, we hypothesized that the persistent symptom queries that were significantly linked to subsequent seeking of information on the 12-step programs would be common or nonsevere in nature; a common or nonsevere symptom would need to be persistent to sufficiently compel a search for addiction treatment information. This third hypothesis was only partially confirmed, as several common and nonsevere symptoms prompted near-immediate search for AA (but not for NA or for NA and AA) information. However, as hypothesized, most persistent and near-persistent symptom queries were about common or nonsevere symptoms (ie, blister, itch, swelling, tinnitus, dizziness, heartburn, and diarrhea for AA and cough, cramp, constipation, fever, and back pain for NA).

Notably, some symptoms were vague regarding severity. Specifically, regarding blurred vision for AA, pain and weight loss for NA, and pain for NA and AA, the extent of pain and weight loss were not specified and blurred vision could potentially range from mild (yet persistent) to severely impairing vision loss. Queries about the vague symptom of “deformity” was also nearly persistent for AA information seekers, which may reflect concerns about appearance with increasing alcohol misuse but may also reflect concerns about pregnancy and alcohol-related birth defects.

In contrast, symptoms such as hallucination, akathisia, amnesia, blindness, and cataplexy (ie, the brief loss of voluntary muscle movement) can more clearly be categorized as severe, although in the absence of comprehensive psychiatric interviews, it was not possible to discern whether these symptoms reflected a primary medical condition/psychiatric disorder or were secondary to substance intoxication, substance withdrawal, or the adverse effects of psychiatric medications (see Limitations).

**Symptom queries preceding the first search for information on the 12-step programs. Medical symptoms and conditions ranked from the highest to lowest according to the ratio of the probability that the symptom will be queried by those who later search for information on the 12-step programs, compared to the rest of all United States Bing users. A Chi-square test found no significant differences between medical symptom queries that preceded searches for information on the 12-step programs versus meeting-specific searches for the 12-step programs. For symptoms appearing in both general and meeting-only categories, the differences in the number of searches is statistically significant.**

**General or meeting information**

Queries for alcoholics anonymous information

- Exophthalmos (bulging eyes)
- Pyrosis (heartburn)
- Hallucination
- Impotence
- Back ache
- Urticaria (hives)
- Akathisia (inner restlessness, inability to stay still)
- Diaphoresis (heavy sweating)
- Agoraphobia
- Cataplexy (brief loss of voluntary muscle movement)

Queries for narcotics anonymous information:

- Pyrosis (heartburn)
- Hallucination
- Impotence
- Urticaria (hives)
- Agoraphobia
- Bloating
- Blindness
- Amnesia
- Dizziness

**Meeting information only**

Queries for alcoholics anonymous information:

- Exophthalmos (bulging eyes)
- Pyrosis (heartburn)
- Hallucination
- Impotence
- Back ache
- Akathisia (inner restlessness, inability to stay still)
- Urticaria (hives)
- Diaphoresis (heavy sweating)
- Agoraphobia
- Amnesia

Queries for narcotics anonymous information:

- Bloating

**Table 2:** Medical symptom search increasing in a statistically significant manner over the 30 days preceding seeking of information on the 12-step programs.

Symptom persistence	Category of the 12-step treatment information sought		
	Alcoholics anonymous	Narcotics anonymous	Both
Persistent (manifesting at all three time points)	Anxiety^a,b,c^ Blister^a,b,c^ Blurred vision^a,b,c^ Itch^a,b,c^ Swelling^a,b,c^ Tinnitus (ears ringing)^a,b,c^	Anorexia^a,b,c^ Cough^a,b,c^ Cramp^a,b,c^ Depression^a,b,c^ Pain^a,b,c^ Weight loss^a,b,c^	Pain^a,b,c^
Near persistent (manifesting at two of the three time points)	Dizzy^a,b^ Deformity^a,b^ Pyrosis (heartburn)^a,b^ Paresthesia (skin crawling)^a,b^ Diarrhea^b,c^ Malaise^b,c^ Phobia^b,c^ Tired^b,c^ Depression^a,c^	Constipation^a,b^ Fever^a,b^ Back pain^b,c^	Anxiety^b,c^
Immediately before 12-step information seeking (manifesting at the last time point only)	Cramp^c^ Hallucination^c^ Phobia^c^ Rash^c^ Toothache^c^ Xerostomia (dry mouth)^c^	—^d^	—
Distal to 12-step information seeking (manifesting at the first time point only)	Dysphagia (trouble swallowing)^a^	Paranoia^a,b^ Perspiration^a^ Diarrhea^b^	Paranoia^a^

^a^Symptom search occurred on days 0-7 in the 30-day lead up to a 12-step program information query on day 30.

^b^Symptom search occurred on days 7-14 in the 30-day lead up to a 12-step program information query on day 30.

^c^Symptom search occurred on days 14-30 in the 30-day lead up to a 12-step program information query on day 30.

^d^None.

## Discussion

### Principal Results

The present study used anonymous Web-search log data analysis to examine which medical symptom queries best motivated subsequent searches for the general and meeting information on the 12-step programs. Our results suggest that queries about more severe or dangerous medical consequences as well as more nonsevere or common symptoms were important in motivating individuals toward subsequently seeking information on the 12-step programs.

Our findings support past work demonstrating the high prevalence of co-occurring psychiatric disorders in addiction [60,61] and the current knowledge base supporting the efficacy of physician advice about severe long-term medical consequences in motivating individuals towards addiction treatment options [62].

These results underscore an important point: Our results do not support the notion that more severe, long-term medical consequences of drugs and alcohol should, in any way, be ignored during brief advice by a physician, but rather, that adding on to current BI practices with additional emphasis on common nonsevere symptoms may be additionally beneficial.

### Potential Underlying Mechanisms

Our findings raise questions about the possible underlying mechanisms that might make common or nonsevere medical symptoms persuasive for people to seek information on 12-step programs. One possible underlying mechanism is that more common symptoms may better allow individuals to envision the negative medical consequences of AUDs and SUDs compared to more chronic or dangerous symptoms. In other words, since a symptom like heartburn is common, it may be easy to identify with the experience of the symptom, notice it worsening with increasing substance use, and envision it becoming even more persistent or chronic. In contrast, it may be difficult for an individual to envision obtaining a cancer diagnosis or its likelihood may be discounted as too distant for concern; moreover, for those who can envision a cancer diagnosis, such envisioning may be so frightening that it fosters denial rather than motivation. Emphasizing the potential for worsening frequency and discomfort of otherwise common symptoms (eg, heartburn, dizziness, hives, and back ache) in addition to severe medical consequences could help further motivate individuals to accept treatment after being newly identified in primary care settings as having an AUD or SUD.

A second possible mechanism underlying our findings may be related to the social embarrassment associated with certain medical symptoms. For example, symptoms such as bloating, bulging eyes, and impotence may impact physical attractiveness. Social conformity has been shown to be an important motive for alcohol and drug use [63], with alcohol, in particular, being considered a social lubricant [64]. Adding to BI’s current practices, highlighting how the medical consequences of substance use can be socially embarrassing may also help motivate patients newly diagnosed with AUD or SUD to consider treatment options including 12-step programs. Although discussion of the social repercussions of drinking alcohol is already a component of the brief motivational interviewing that is sometimes incorporated into BI, our results suggest it may provide additional motivation to discuss how medical and nonmedical consequences can interact to intensify social embarrassment in patients with newly identified AUD or SUD.

In sum, our results suggest that many common or nonsevere medical symptoms and conditions motivate subsequent engagement with 12-step programs. As a result, BI could be restructured in multiple ways to maximize patients’ motivation to engage in treatment, including accompanying current BI practices and highlighting the common medical symptoms that could immediately worsen with increasing substance misuse and how some of those worsening symptoms could exacerbate social embarrassment. Such findings may help inform SBIRT efforts to screen individuals who present with medical complaints for AUD, provide brief interventions that motivate individuals toward considering treatment options, and provide referrals to AUD treatments. Specifically, our findings suggest that it may be beneficial to modify SBIRT’s BIs to add focus on the contribution of alcohol to immediately discomforting and socially embarrassing medical symptoms.

### Limitations

A primary limitation of the present study was the anonymous nature of Web-search log data, which did not allow us to collect information on multiple demographic factors including age, sex, ethnicity, and socioeconomic status. Another primary limitation was our inability to follow-up on querying behavior to gauge whether search queries translated into real-world action. For example, although someone in the Prochaska and DiClemente [65] precontemplation stage of change would be very unlikely to run a search query for “AA information” (and even more unlikely to query for “AA meeting near me”), we cannot be certain whether search queries denote a contemplation, preparation, or action stage of change. In other words, we were unable to determine if searching for “AA meeting near me” was a query soon followed by AA meeting attendance or whether it was only gathering information for those contemplating or preparing to make a change but not yet ready to take action.

We also noted that in a minority of cases, people may be searching for others and multiple people may be using the same browser, thus conflating multiple people to the same user identifier. However, although this unlikely possibility cannot be ruled out entirely, it is estimated based on past work [66] that these searches are limited to only a small fraction of the data. For instance, although the keyword “alanon” was included, which typically reflects family members searching for loved ones, we found that only 1.4% of queries we studied contained this keyword.

Our study also lacked comprehensive psychiatric interviews, and thus, it was not possible to discern if certain symptoms were suggestive of co-occurring psychiatric conditions, the adverse side effects of psychiatric medications, substance-withdrawal symptoms, severe medical consequences of substance misuse, the use of multiple substances, physical comorbidities related to the searched-for symptoms, or the social consequences of excessive drinking. For instance, symptoms like hallucination and akathisia could reflect substance withdrawal (ie, withdrawal-related hallucinations and severe agitation) or a co-occurring psychotic disorder alongside the adverse effects of the antipsychotic medication used to treat it. Similarly, phobia queries may signify a co-occurring anxiety disorder or withdrawal-related anxiety increases. Further, queries about amnesia may reflect concerns about blackout drinking, withdrawal-related states of delirium, or the severe cognitive repercussions of long-term substance misuse (ie, memory loss).

Our anonymous online search data also grouped all people with NA queries together, and thus, our data did not allow us to differentiate between subgroups of narcotics being misused. For example, stimulants and sedatives have different and often opposing medical consequences. Thus, we did not have access to data that could have improved our precision in delineating the medical symptoms that preceded NA information queries.

Our data were exclusively derived from Bing searches and did not include data from other search engines such as Google or Yahoo. Future studies are needed to replicate our findings in other search engines as well as at a smaller scale where demographic characteristics can be more comprehensively collected (and statistically compared and contrasted) rather than estimated based on profoundly large usage data.

Our study scope was limited to an investigation of how delay discounting may impact online treatment information seeking, and indeed, there are many barriers to addiction treatment beyond delay discounting that our study did not investigate. This limited scope reflected our need to maintain study focus. Future studies are needed to expand on other potential barriers. Further, the conceptual scope of our study was also limited by the technical limitations of online search data analysis, which relies on an adequately large number of single keywords or short phrases that are well defined and unambiguous in their meaning and ability to be linked together. First, our treatments were limited to twelve step programs without other forms of evidence-based treatment such as counseling or medication. A prerequisite of studying online search data is that any chosen query sets must be reasonably likely to have sufficient public visibility to gather a large enough number of responses for analysis. As a result, we narrowed our scope to 12-step programs, the most highly utilized form of addiction treatment [67] and therefore the most likely to have the greatest number of search queries. Second, we limited our predictors to adverse medical symptoms without other kinds of addiction-related problems such as social or legal consequences. Online search data analysis is not yet able to adequately capture more nuanced metrics such as social and legal problems without a significant number of false positives. To address this limitation, we opted to solely focus on medical symptoms because of pre-existing mapping of Web-search queries to medical symptoms that in the past work, has successfully limited false positives and avoided off-base random exploration [68]. Although accommodating these technical limitations narrowed our study focus, elucidating how medical symptoms impact seeking information on the 12-step program can offer unique insights critical to SBIRT and other early intervention efforts in primary care settings. Nevertheless, future studies are needed to expand upon the scope of our line of research inquiry to include other forms of treatment and consequences of addiction.

### Conclusions

In conclusion, our anonymous search log data analysis indicated that querying about the 12-step program was preceded by the occurrence of a number of nonsevere medical consequences of alcohol and drug misuse. The persistence of these nonsevere symptoms also appeared to play an important role in motivating individuals to query about the 12-step program. These findings can help inform modifications to current SBIRT protocols by concurrently emphasizing the long-term medical consequences of alcohol and drug misuse alongside shorter-term sets of symptoms that are more immediately discomforting and socially embarrassing. Such modified SBIRT protocols may apply BI to improve the motivation of the ~17% and 22.7% of individuals who screen positively during primary care encounters (using brief screening instrument scores) for risky/problematic alcohol and drug use, respectively [69,27] in order to accept referrals to 12-step programs or other outside addiction-treatment resources.

## Appendix

Multimedia Appendix 1 Alcoholics Anonymous and Narcotics Anonymous webpages queried using the Bing search engine.

## Abbreviations

AA: alcoholics anonymous
AUD: alcohol use disorder
BI: brief intervention
NA: narcotics anonymous
SBIRT: Screening, Brief Intervention, and Referral to Treatment
SUD: substance use disorder
