# Supplementary material for: Internet Searches for Medical Symptoms Before Seeking Information on 12-Step Addiction Treatment Programs: A Web-Search Log Analysis
Source: J Med Internet Res. 2019 May 3;21(5):e10946. doi: 10.2196/10946 (PMC6533047; doi:10.2196/10946)
Supplement: Multimedia Appendix 1 [file jmir_v21ie10946_app1.pdf]

Multimedia Appendix 1. AA and NA webpages queried using the Bing search engine

http://www.aa.org/
http://www.aa.org/pages/en_us/find-local-aa
https://na.org/
http://www.aa.org/pages/en_us/daily-reflection
http://www.na.org/
http://www.naws.org/meetingsearch/
https://www.na.org/?id=home-content-fm&id=home-content-fm
http://www.aa.org/pages/en_us/find-aa-resources
http://www.aa.org/pages/en_us/read-the-big-book-and-twelve-steps-and-twelve-traditions
http://www.aa.org/pages/en_us/alcoholics-anonymous
http://www.jftna.org/jft/
http://www.na.org/?id=home-content-fm
http://www.coastalcarolinaarea.org/literature/books/swg.pdf
http://alcoholicsanonymous.com/
http://www.the-alcoholism-guide.org/alcoholic-anonymous-12-steps.html
https://aa-meetings.com/
http://www.recovery.org/topics/alcoholics-anonymous-12-step/
http://12step.org/references/12-step-versions/na/
http://alcoholicsanonymous.com/find-a-meeting/
http://www.aa.org
https://www.na.org/?id=literature&id=literature
http://www.aaonline.net/
https://www.na.org/index.php?id=home-content-fm&id=home-content-fm
http://www.na.org/index.php?id=home-content-fm
http://www.na.org/meetingsearch/
http://www.nanj.org/jft2.shtml
http://nnjaa.org/
http://m.na.org/
http://www.aa.org/pages/en_us/information-for-aa-members
http://www.aa.org/assets/en_us/smf-121_en.pdf
http://coastalcarolinaarea.org/literature/books/b_t.pdf
http://na-recovery.org/narcotics_anonymous_12_steps.html
http://www.aa.org/pages/en_us/find-aa-resources?zipcode=zip%2fpostal+code
http://www.aa.org/pages/en_us/what-is-aa
http://www.al-anon.alateen.org/
http://na-recovery.org/narcotics_anonymous_online_meeting_schedule.html
http://webdata.na.org/events/
http://www.aa.org/pages/en_us/twelve-steps-and-twelve-traditions
http://www.aahouston.org/
http://www.aasandiego.org/
http://www.namiddlesexnj.org/jft
http://www.na.org/?id=literature
http://www.nar-anon.org/
http://www.alcoholics-anonymous.org.uk/about-aa/the-12-steps-of-aa

http://primarypurposearea.org/just_for_today.htm
http://www.aa.org/assets/en_us/alcoholics-anonymous/b-1-alcoholics-anonymous
http://stopaddiction.com/12-step/12-steps-of-narcotics-anonymous
http://www.cleanandsobernotdead.com/meeting%20quotes/meeting%20quotes.html
http://alcoholicsanonymous.com/aa-meetings/massachusetts/
http://thesponsorsaide.org/step1.htm
http://www.naohio.org/
https://www.na.org/?id=litfiles/us_english/misc/just%20for%20today.pdf&id=litfiles/us_english/misc/just%20for%20today.pdf
http://michigan-na.org/
http://na-recovery.org/internet_sources_of_narcotics_anonymous_literature.html
http://www.recovery.org/topics/about-the-narcotics-anonymous-na-12-step-recovery-program/
https://www.na.org/admin/include/spaw2/uploads/pdf/handbooks/igg.pdf
http://aaneworleans.org/
http://www.aa.org/pages/en_us/communication-with-the-general-service-office-gso-of-aa-in-new-york-city
http://www.recovery.org/topics/alcoholics-anonymous-the-big-book/
http://aaphoenix.org/
http://www.step12.com/step-4.html
https://aa-san-mateo.org/
http://www.recoverytimes.com/terry8.html
https://www.scientificamerican.com/article/does-alcoholics-anonymous-work/
http://alcoholicsanonymous.com/aa-meetings/pennsylvania/
http://arizona-na.org/meetings/valley.php
http://printableworksheets.in/?dq=narcotics%20anonymous
http://www.aa.org/pages/en_us/aa-literature
http://www.aa.org/pages/en_us/alcoholics-anonymous-audio-version
http://www.aaboston.org/
http://www.daccaa.org/meetings.htm
http://www.pdfsmanualsbook.com/narcotics-anonymous-printable-step-working-guide.pdf
http://alcoholicsanonymous.com/aa-meetings/california/
http://alcoholicsanonymous.com/aa-meetings/texas/
http://www.aa.org/pages/en_us/aa-timeline
http://www.justfortodaymeditations.com/tag/daily-meditations-aa/
http://www.simeetings.com/la/lamtgs.html
https://www.goodreads.com/author/quotes/2914435.alcoholics_anonymous
https://www.na.org/?id=ips-eng-index&id=ips-eng-index
http://aaminneapolis.org/
http://alcoholicsanonymous.com/aa-meetings/florida/
http://atlantaaa.org/
http://na-recovery.org/
http://www.aa-semi.org/
http://www.aastpaul.org/
http://www.na.org/?id=ips-eng-index
http://12step.org/tools/12steporg-worksheets/
http://alcoholicsanonymous.com/aa-meetings/ohio/

http://alcoholicsanonymous.com/aa-meetings/wisconsin/
http://my12stepstore.com/
http://www.ct-aa.org/
http://www.sierrasagena.org/
https://b2c.aaws.org/
http://alcoholicsanonymous.com/aa-meetings/illinois/
http://austinaa.org/
http://ctana.org/
http://pcana.org/
http://portlandna.com/
http://www.aa.org/assets/en_us/en_step1.pdf
http://www.aageorgia.org/
http://www.jftna.org/jft/index.php?vm=r&s=1
http://www.lvcentraloffice.org/
http://www.namontana.com/
https://aastl.org/
https://www.aadallas.org/wordpress/
https://www.na.org/?id=bt6e_webposting.pdf&id=bt6e_webposting.pdf
http://aasacramento.org/
http://aatampa-area.org/
http://home.onemain.com/~gpg/na_baby_blue_book.pdf
http://www.aamilwaukee.com/
http://www.alcoholic.org/research/aa-step-1/
http://www.chicagona.org/
http://www.justfortodaymeditations.com/tag/na-just-for-today/
http://www.sandiegona.org/
https://brainly.com/question/1385651
https://coloradoaa.org/
https://www.addiction.com/meetingfinder/
https://www.chicagoaa.org/
https://www.na.org/admin/include/spaw2/uploads/pdf/litfiles/us_english/misc/how%20it%20works.pdf
https://www.nerna.org/
http://aasantacruz.org/meeting-schedule.aspx
http://choopersguide.com/content/na-narcotics-anonymous-meeting-format.html
http://daccaa.org/
http://kc-aa.org/
http://na-hawaii.org/
http://nawol.org/2012_12princ.htm
http://wisconsinna.org/
http://www.aa.org/assets/en_us/en_step4.pdf
http://www.aa.org/pages/en_us/aa-general-service-board-calendars
http://www.justfortodaymeditations.com/
http://www.naminnesota.org/
http://www.nar-anon.org/find-a-meeting
http://www.ny-aa.org/

https://ks-aa.org/
https://www.amazon.com/big-book-alcoholics-dr-smith/dp/1483907252
https://www.amazon.com/just-today-meditations-recovering-addicts/dp/1557761515
https://www.ct-aa.org/find-a-meeting/
https://www.okna.org/
https://www.rochester-ny-aa.org/
https://www.seattleaa.org/
http://aahouston.org/locator/index.php?tab=us
http://aainthedesert.org/
http://aamesaaz.org/
http://alcoholicsanonymous.com/aa-meetings/maryland/baltimore/
http://everettwana.org/
http://foundersdayregistration.akronaa.org/
http://naflorida.org/
http://www.idahoarea18aa.org/
http://www.larna.org/
http://www.naindiana.org/showarea.php?iareaid_req=2
http://www.naminnesota.org/index.php/meeting-list-pdf
http://www.narcoticsanonymousnj.org/meetings/
http://www.natucson.org/meetinglist.html
https://na.org/?id=home-content-fm
https://www.na.org/?id=litfiles/us_english/misc/just%20for%20today.pdf
https://www.na.org/?id=orderliteratureonline-naw&id=orderliteratureonline-naw
https://www.na.org/?id=phoneline&id=phoneline
https://www.na.org/admin/include/spaw2/uploads/pdf/litfiles/us_english/ip/en3110.pdf
http://aa52centraloffice.org/
http://aanashville.org/
http://aasantacruz.org/
http://aatricitiestn.org/images/thetry_this_12-steps_and_12_traditions-1.pdf
http://aatucson.org/
http://alcoholicsanonymous.com/aa-meeting/
http://ctna.org/
http://m.bklynna.org/
http://oc-aa.org/directory/meetings.asp?day=select+a+week+day&city=select+a+city
http://sacramentona.org/
http://www.12step.org/references/12-step-versions/aa/
http://www.aa.org/assets/en_us/en_step2.pdf
http://www.aa.org/assets/en_us/smf-56_en.pdf
http://www.aa.org/pages/en_us/daily-reflection?y=2016&m=09&d=19
http://www.aacleve.org/
http://www.aanatcon2017.com.au/
http://www.coastalcarolinaarea.org/literature/books/h_w.pdf
http://www.fresnoaa.org/
http://www.grandrapidsaa.org/
http://www.royy.com/step4.pdf
https://aavirginia.org/

https://todayna.org/
https://www.na.org/?id=ips-eng-index
https://www.na.org/?id=wcna-index&id=wcna-index
